# The Book World of Medicine and Science

**Published:** 1904-10-29

**Authors:** 


### The Book World of Medicine and Science.

A Manual of Practical Medical Electricity. By Dawson Turner, B.A., M.D., F.R.C.P.Ed. Fourth Edition. (London: Baillière, Tindall and Cox. 1904. Pp. 435. Illustrations 200. Price 10s. 6d. net.)

This, the fourth edition of Dr. Turner's text-book, has been considerably enlarged by the insertion of additional matter dealing chiefly with the therapeutic effects of radium and high-frequency currents. The contents of the volume may be divided into two portions, dealing with electro-physics and electro-therapeutics respectively. The treatment of these is singularly unequal, and it is from the former that the reader will derive most information. The descriptions of the apparatus necessary for the generation of the static, galvanic, and faradic forms of electricity are clear, practical, and well illustrated by diagrams. Equally practical is the advice as to the choice of apparatus and the best means of adapting it for various purposes, and these chapters may be commended as a guide to those contemplating an electric installation for medical purposes. Passing by the section on electro-diagnosis, which adds little to what is already known on the subject, we come to the therapeutic applications of electricity. Here the plan followed has been to take up the various conditions in which electricity has been employed, and to describe briefly the methods made use of by various writers for their relief. The result is a somewhat confusing mass of information, which is presented to the student without much help from the author, who rarely indicates his personal experience of the various procedures. In the chapters on Roentgen rays, Finsen light, radium, and high-frequency currents, the practical directions of the methods employed are in striking contrast to the descriptions of the results obtained, which are often marred by a vagueness of statement. While the therapeutic value of Roentgen rays and Finsen light are definitely established, the author regards the effects of high-frequency currents as still upon trial, and we agree with Dr. Turner that if these newer physical methods of treatment are to be put on a scientific basis, it must be by eliminating the lay electrician and retaining their application in the hands of the medical profession.

The Story of an East London Hospital. (London: Macmillan and Co. 1904. Price 2s. 6d. net.)

This little volume tells the story of the foundation of the East London Hospital for Children. In doing so, it gives the history of two heroic lives, those of Dr. Nathaniel Heckford and his wife. These two met when Heckford was a young physician at the London Hospital, and was helping to subdue a cholera epidemic at Wapping, where Miss Sarah Goff had gone to work as a volunteer nurse. Sympathy of mind developed into affection, and early in 1867 the two young workers were married to each other. Then, says Mrs. Heckford, "we determined to work out our theories of life as it ought to be, on thoroughly new principles; we were somewhat undetermined as to what to do, but we agreed that we must do something to show how much happiness might ensue if persons of means and culture would devote themselves to elevating those less fortunate than they." Finally they resolved to start a children's hospital, which was opened on the first anniversary of their wedding day. Dr. Heckford died at the age of twenty-nine, leaving, however, the hospital as his permanent memorial. His widow survived for more than thirty years—years full of original philanthropy. At Naples, where she lived for some years, she devoted herself to a crusade against the cruelty to the lower animals which she saw everywhere around her; then she went to India, travelled much in unfrequented parts, and finally became medical attendant to an Indian Begum. Her last philanthropic efforts were made in the Transvaal where she tried to form a "Transvaal Education Union." The book is worth possessing if only for the story of Nathaniel and Sarah Heckford.

Practical Chemistry. By P. A. Ellis Richards, F.I.C., Professor of Chemistry at Queen's College, London. (London: Baillière, Tindall and Cox. 1904. Pp. 136. Price 3s. net)

This handy little manual of practical chemistry is arranged to meet the requirements of the Conjoint Board Preliminary Science Examination as recently revised and also the Preliminary Scientific Examination of London University. In addition to the usual tables for the detection of metals and acids and directions for the preparation of some of the simple salts, an excellent section is given on elementary volumetric analysis. Here the underlying principles are clearly explained, while the preparation and use of the standard solutions and the calculation of results are well described and illustrated by examples. A novel feature is the addition of a section on elementary practical toxicology in which the reactions of the common poisons are described and also the method by which an organic mixture may be systematically examined with a view to their detection. Clear instructions on this subject are of great practical importance, and these will prove of value after the earlier portions of the book have lost their interest.

Laws of Health. (London: McDougall's Educational Company, Ltd. 8vo. Pp 80. Price 6d. net.)

This is designed as a school text-book or reader for senior classes. In addition to elementary physiology it contains chapters on air, water, food, ventilation, drainage and the spread of disease, with instruction in first aid. While the information is excellent of its kind it is not always presented in a form sufficiently attractive to the class of reader for whom it is intended. The condensation of so many intricate subjects in a limited space has led to the retention of technical terms which might otherwise have been avoided. A number of well-chosen illustrations help to explain the structure of the body and the methods of ambulance work.

The Excursions of an Improvident Philosopher. By Y. H. C. P. Wanhope, M.A. (Norwich: A. H. Goose. 1904. Pp. 115. Price 1s. net.)

This forms a little volume of holiday sketches reminiscent of pleasant days in Egypt and the Near East. Entirely free from formal description, the minor incidents of travel are presented with a lightness of touch, combined with an acuteness of observation, which makes entertaining reading, and there is just sufficient medical allusion to leave no doubt as to the author's profession. His impressions of Eastern Europe, and especially of the relations of the Russian Government and priesthood to the peasantry, are of interest at the present moment.

